# NL-YOLOv5: a model with a larger receptive field and the ability to globally acquire features

**DOI:** 10.3389/fnbot.2026.1764856

**Published:** 2026-05-29

**Authors:** Zhiyu Li, Jinhu Liu, Zhihao Zhuo, Lin Chen, Xiao Zeng, Di Li, Yongfa Zhou, Chunzhou Huang

**Affiliations:** Guangdong Power Grid Company Zhuhai Electric Power Supply Bureau, Zhuhai, Guangdong, China

**Keywords:** DCGAN, deep learning, landslide, NL-YOLOv5, object detection

## Abstract

**Introduction:**

Landslide disasters cause severe casualties and economic losses, demanding rapid and accurate detection from high-resolution remote sensing imagery. Traditional methods struggle with insufficient landslide samples and low detection accuracy.

**Methods:**

To address this, we propose a dual solution: (1) DCGAN-based data augmentation generating 3,429 synthetic landslide samples from Google Earth imagery, significantly improving model generalization; (2) an NL-YOLOv5 model integrating non-local attention (NLA) and an improved LK-SPP module (based on large-kernel convolution concepts) to enhance global information capture.

**Results:**

The NL-YOLOv5 model achieves 80% detection accuracy (a 5% improvement over baseline), with 7% higher precision, 2% higher recall, and 5% higher F1-score, while maintaining real-time speed at 69 f/s.

**Discussion:**

This work delivers a practical solution for high-precision landslide detection in remote sensing applications.

## 1 Introduction

Geological disasters include landslides, collapses, debris flows, ground subsidence, etc. Among them, landslide disasters are one of the most severe geological disasters causing casualties and economic losses in China and even worldwide (Feng et al., 2026). Landslides occur as a geological phenomenon of surface rock mass movement when the slope cannot withstand its own weight and external forces due to external disturbances (Bui et al., 2025). There are many factors triggering landslide geological disasters, mainly including earthquakes, heavy rainfall, surface water erosion, and unreasonable human activities. For example, in September 2022, a magnitude 6.8 earthquake occurred in Luding County, Ganzi Prefecture, Sichuan Province, triggering large-scale landslides in the region and causing severe losses of life and property to local residents (Zhu et al., 2025; Du et al., 2025). At the same time, traffic congestion caused by landslides brought great difficulties to emergency rescue efforts. Landslide disasters not only cause losses of life and property but also have varying degrees of impacts on the social economy and surface environment (Zhou et al., 2025). According to statistics, there were 2,335 landslide disasters in China in 2021 alone. Rapid and accurate acquisition of landslide locations, volumes, extents, and other information after landslides occur and timely preparation of landslide distribution maps are of great significance for post-disaster emergency rescue, disaster loss assessment, landslide change monitoring, and landslide treatment (Fang et al., 2025). On the other hand, according to disaster information released by the Ministry of Natural Resources, most new landslides in recent years have occurred outside the cataloged landslide areas. Therefore, analyzing the causes and controlling factors of landslides after they occur, identifying existing landslides, and establishing detailed landslide catalogs are of great value for the development and evaluation of landslide models (Cuomo et al., 2025).

In recent years, with the influence of various extreme climates, the frequency of landslide disasters has been increasing. Therefore, research on landslide disaster monitoring and early warning and post-disaster rapid identification and detection technologies is urgent. Currently, landslide detection methods can be roughly divided into three categories based on different levels of automation: (1) Traditional manual visual interpretation methods. These methods are highly subjective and have slow identification speeds, so they can no longer be the mainstream application methods in the context of massive remote sensing imagery data (Kumar et al., 2025; Li et al., 2025). (2) Semi-automatic identification methods with human-computer interaction. These methods mainly use computer identification combined with visual interpretation, designing landslide features through visual interpretation and then using computer algorithms to achieve landslide extraction (Chen et al., 2022). This type of semi-automatic identification method with human-computer interaction has a significantly improved identification speed compared to visual interpretation methods, but the manual design of landslide identification rules still makes the automation level of such methods insufficient. (3) Automatic methods with self-learning features. Deep learning (LeCun et al., 2015) methods are currently the better automatic methods applied in automatic landslide identification. Compared to the first two methods, this method completely gets rid of the cumbersome operation of manually designing features by automatically extracting landslide features through convolutional neural networks, thus greatly improving the automation level of landslide identification (Wang et al., 2024).

With the further development of remote sensing technology, China has also entered the era of high-resolution remote sensing, and a massive amount of high-resolution remote sensing imagery will be transmitted to the ground every day in the future. Facing future remote sensing big data, traditional remote sensing landslide interpretation methods, due to their low automation levels, have been unable to meet the interpretation work of massive remote sensing data. Therefore, how to make use of the self-learning feature characteristics of deep learning to solve the problem of high-precision real-time detection of landslide disasters has become a research focus.

## 2 Related work

(1) In recent years, deep learning methods have also been widely applied in the field of landslide detection. From traditional methods based on topographic data to the gradual introduction of multi-source data fusion methods combining remote sensing imagery and meteorological data, and then to more complex neural network structures and more refined data processing methods in recent years. The development of these methods can not only improve the accuracy of landslide detection and prediction but also provide more precise support for the prevention and treatment of landslide disasters. Similarly, deep learning-based landslide detection methods are also constantly evolving with the continuous improvement of deep learning theories. From 2018 to 2020, the application of deep learning methods in the field of landslide detection began to develop. As the depth of the network increased, the features acquired by the model became more abstract and unique, thus further improving the model detection accuracy. Researchers summarized previous experiences and began to design and use models with more complex network structures. Ghorbanzadeh et al. (2019), in order to verify whether convolutional neural networks are necessarily superior to traditional machine learning models in the field of landslide remote sensing detection, analyzed the performance of traditional machine learning methods and shallow convolutional neural networks for landslide detection using optical remote sensing imagery and topographic data. The authors constructed 7-layer and 5-layer CNN landslide detection models and conducted comparative experiments with machine learning algorithms such as artificial neural networks, support vector machines, and random forests, concluding that the detection performance of CNN models is not necessarily stronger than that of traditional machine learning algorithms, and their performance largely depends on network design, i.e., network depth, input window size, and training strategies. With the development of deeper CNN networks, some researchers began to use more complex models for landslide detection research, among which two-stage CNN models were widely applied (Du et al., 2020). Zhang et al. (Wenyue et al., 2023), in order to retain useful feature information to a greater extent, effectively avoided the interference of maximum values on the results by changing the pooling type from max pooling to random pooling in the model. To effectively identify and locate small targets in remote sensing imagery, several small-sized anchors were added to the network model. Based on the analysis and research of various convolutional neural network models and related parameters, an improved Faster R-CNN (Girshick, 2015; Ren et al., 2015) landslide detection model was proposed according to the characteristics of landslide target detection. This model belongs to a two-stage deep complex network and achieved good detection results when applied to landslide target detection. Zbigniew et al. (Omiotek, 2025), aiming at the problems that traditional landslide identification methods mainly rely on human effort, existing automatic landslide identification methods are mainly based on machine learning, and landslide data sources rarely use Google Earth imagery, established a historical loess landslide sample database based on Google Earth imagery and used the two-stage instance segmentation network Mask R-CNN (He et al., 2017) target detection module to accurately identify loess landslides in Lanzhou City and Linxia Hui Autonomous Prefecture, Gansu Province. From the research results of this stage, it can be seen that the application of deep learning methods in the field of landslide detection has been more widely developed. Researchers are more inclined to use convolutional neural network models with deeper networks and more complex structures for landslide detection research. However, these methods still have some limitations, such as poor model interpretability and excessive model parameters that cannot well serve emergency rescue after landslide disasters. From 2021 to the present, deep learning-based landslide detection methods have become more widespread, and more new technologies and methods have emerged. Due to the timeliness requirements of landslide disaster emergency rescue detection, more and more researchers have begun to use one-stage target detection algorithms for landslide disaster detection (Wang et al., 2021; Li and Li, 2022). Compared with two-stage algorithms, one-stage landslide detection algorithms have inherent advantages in detection speed, although they are slightly weaker in accuracy (Kumar et al., 2022). Cheng et al. (Cheng et al., 2021) improved on the one-stage target detection network YOLOv4, using phantom residual modules and grouped convolutions to replace ordinary convolution modules to reduce the model’s parameter quantity. At the same time, an attention mechanism was added to the model structure, and a small-parameter landslide disaster detection model YOLO-SA was proposed. The YOLO-SA model was used to quickly and accurately identify landslide disasters in Qiaojia County and Ludian County, Yunnan Province. In this stage, different forms of attention mechanisms were also widely applied in landslide target detection algorithms. Jin and Wang (2025) used a dual attention mechanism to improve multiple different neural network structures and constructed an optimization algorithm based on convolutional neural networks. Jiang et al. (2022) developed the LRSTTC dataset and used Mask R-CNN with transfer learning to detect new and old landslides along the Sichuan–Tibet corridor; Jiang et al. (2025) proposed AdaptVFMs-RSCD, integrating SAM and CLIP for semantic change detection in multi-temporal imagery. However, both approaches rely on substantial labeled data or large foundation models, limiting their applicability in data-scarce, real-time landslide monitoring scenarios like those in southeastern Chongqing. In recent years, models based on Transformer have begun to be introduced into the remote sensing landslide detection task. Zhang et al. (2023) proposed a hybrid architecture that combines CNN and Transformer, achieving more robust landslide recognition performance on multi-source remote sensing images by integrating local feature extraction capabilities with global attention mechanisms. At the same time, Chen et al. (2024) developed a pure Transformer framework called TransLandslide, specifically designed for large-scale landslide mapping of high-resolution satellite images. It effectively utilizes the self-attention mechanism to capture long-distance dependency relationships of ground features, significantly improving the detection accuracy in complex terrains. Landslide detection experiments were conducted on statistical landslide datasets, and the results showed that convolutional neural networks incorporating a dual attention mechanism had higher landslide identification accuracy compared to single neural networks, and the landslide boundary segmentation results were closer to the real situation. It can be seen that in this stage, researchers have begun to conduct high-precision landslide detection method research from multiple perspectives such as algorithm improvement and enhancing model attention. However, deep learning methods still face some challenges and limitations in the field of landslide detection. Firstly, larger datasets are needed to train and validate algorithms. Secondly, there is a class imbalance problem in landslide detection data, i.e., the proportion of landslide areas to non-landslide areas is unbalanced (Al-Najjar et al., 2021; Chen et al., 2022; Tanyu et al., 2021). This may cause the algorithm to focus too much on certain classes and ignore others. Recent works addressing data challenges in landslide studies include Zhang et al. (2024), who refined landslide boundaries via attention U-Net for susceptibility mapping, and Huan et al. (2023), who established multi-scale factor databases for machine learning-based susceptibility assessment. These approaches focus on classification (susceptibility mapping), while our DCGAN-based augmentation directly targets the detection-specific data scarcity problem. Finally, algorithms in the field of ordinary image detection may not perform equally well in landslide remote sensing detection, and further exploration and research are needed to optimize algorithms and improve landslide detection accuracy. In response to the above issues, we have made the following two contributions:

Task-oriented landslide sample generation via GAN-based data augmentation.

To address the limited availability of labeled landslide samples in remote sensing imagery, we adopt a data augmentation strategy based on Generative Adversarial Networks (GANs). Specifically, we employ a Deep Convolutional GAN (DCGAN) architecture—replacing the fully connected layers in the original GAN with convolutional layers—to better capture spatial structures inherent in landslide features. The DCGAN is trained on our curated landslide dataset over multiple epochs, and the checkpoint yielding the best visual and statistical fidelity is selected to synthesize additional realistic landslide samples. The augmented dataset is then used to train and evaluate our detection model, enabling a systematic assessment of how GAN-based augmentation impacts performance in the context of landslide identification.

(2) Systematic integration of established components for landslide detection.

Building upon YOLOv5, we develop a tailored detection pipeline—referred to as NL-YOLOv5—by integrating two well-established techniques that are particularly suited to the challenges of landslide detection: non-local attention and large-kernel spatial pooling. First, recognizing that landslides often exhibit spatially extended and context-dependent patterns, we incorporate a Non-Local (NL) attention module into the backbone network to enhance long-range dependency modeling without altering the core architecture. Second, to further expand the effective receptive field while preserving feature continuity, we replace the standard SPPF module with a modified Large-Kernel Spatial Pyramid Pooling (LK-SPP) block. This module leverages depthwise separable convolutions with large kernels (e.g., 21 × 21, 31 × 31, 41 × 41) as a dense alternative to dilated convolutions, thereby mitigating the gridding artifacts commonly associated with high-dilation ASPP-like structures. Through controlled ablation studies, we empirically determine the optimal kernel size configuration for LK-SPP under the landslide detection task.

Collectively, our work emphasizes task-specific adaptation and rigorous evaluation of existing deep learning components—rather than proposing a fundamentally new architecture—and demonstrates how their synergistic integration can significantly improve detection accuracy in a real-world geohazard monitoring scenario.

## 3 Method

### 3.1 Overview of the study area and dataset

This study focuses on southeastern Chongqing, China (107°30′–109°30′E, 28°10′–30°30′N), bordering Hubei Province to the north, Guizhou Province to the south, and Hunan Province to the east. The area includes six districts/autonomous counties: Qianjiang, Wulong, Shizhu, Xiushan, Youyang, and Pengshui, covering 19,800 km^2^ (20.60% of Chongqing’s total area) with a population of 3.64 million. The region features a subtropical monsoon climate with annual precipitation >1,200 mm, high vegetation coverage (49.4% forest coverage, 8.3% higher than Chongqing’s average), and complex topography dominated by medium- to low-mountain landscapes (elevation >1,000 m). It belongs to the core area of the Wuling Mountains and a national key ecological function zone, frequently experiencing landslides during the heavy rainfall season (June–September).

The original dataset was built using Google Earth’s open-source imagery, integrating multi-source satellite and aerial data:

*Satellite imagery*: QuickBird (up to 0.61 m resolution) and EarthSat data from DigitalGlobe (USA).

*Aerial imagery*: BlueSky, Sanborn, IKONOS (~1 m), and SPOT5 (~2.5 m) data.

To adapt to model input requirements, all images were cropped into 640 × 640-pixel slices containing landslide zones and background information. 778 landslide samples were collected using three data augmentation strategies via Google Earth’s historical imagery.

**Figure 1:**
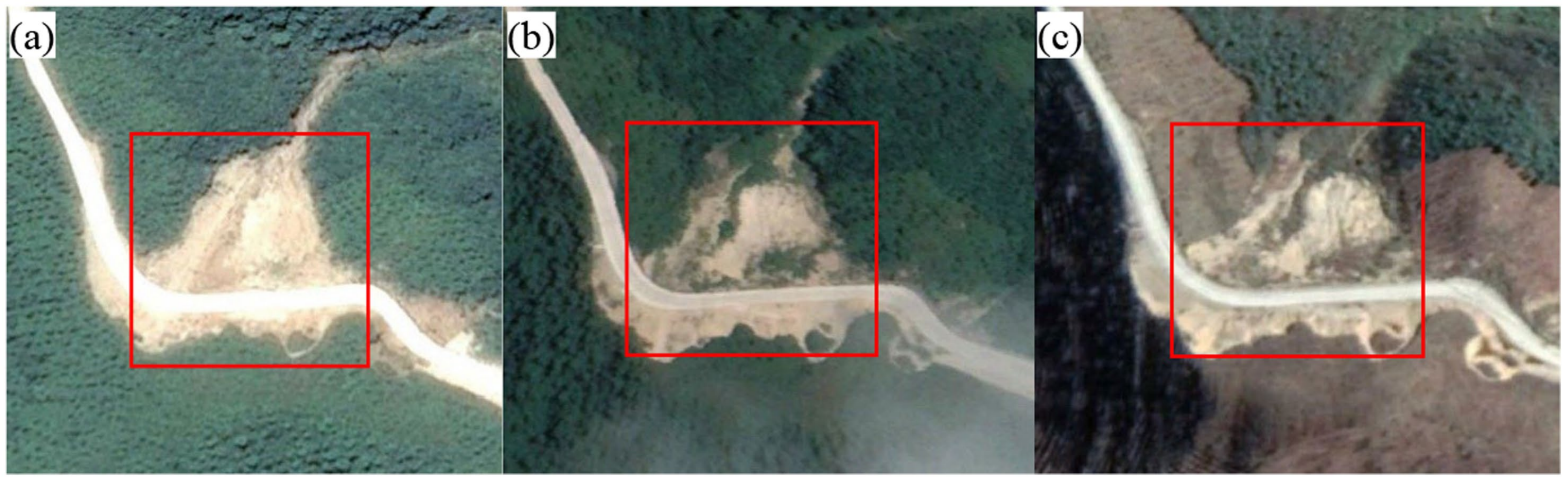
Differences in landslide development.

*Developmental stage differences*: samples captured at different landslide evolution stages (e.g., Figures 1A–C), reflecting shape and texture variations. Among them, the differences are marked with red boxes.

**Figure 2:**
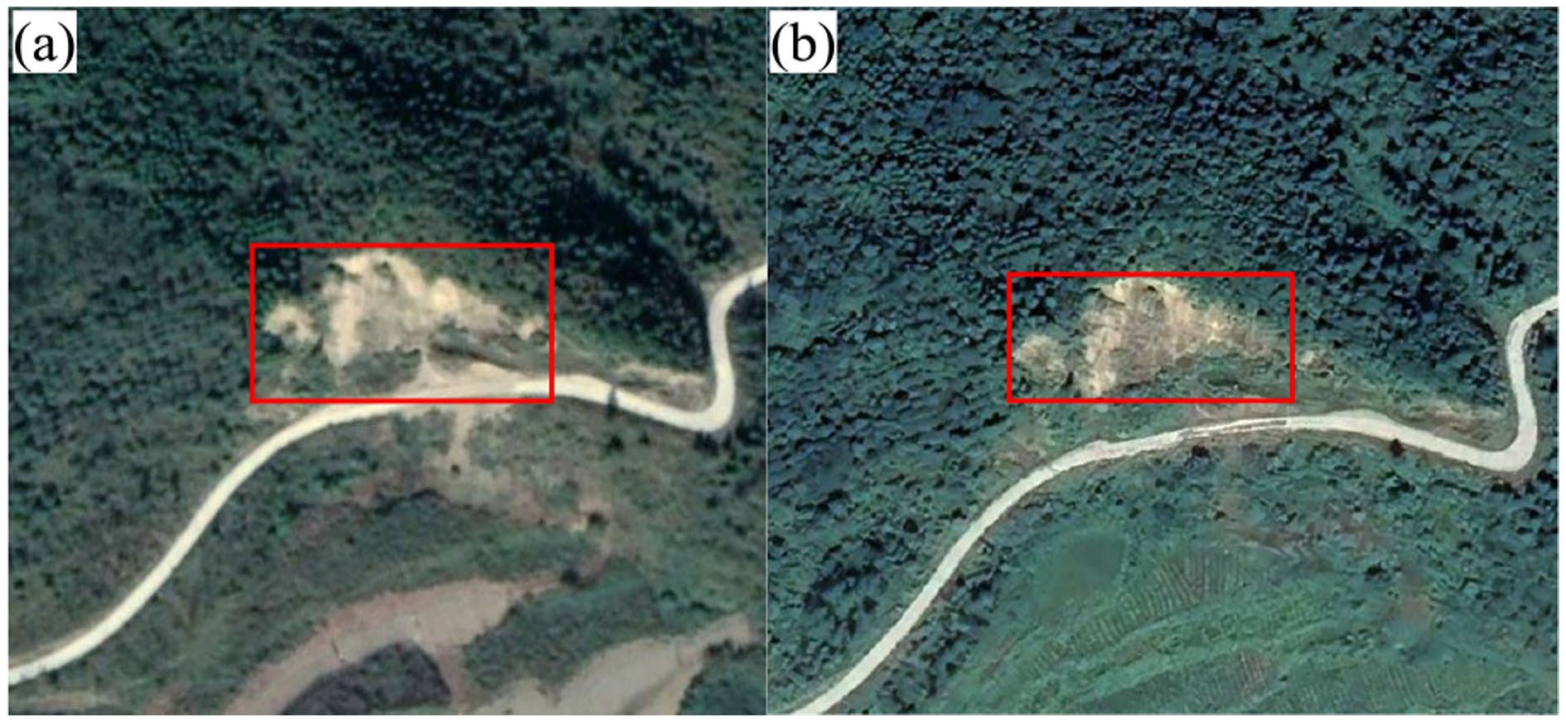
Seasonal differences in landslides.

*Seasonal variations*: seasonal differences in vegetation cover (e.g., dense vegetation in August vs. exposed areas in November, Figures 2A–B). Among them, the differences are marked with red boxes.

**Figure 3:**
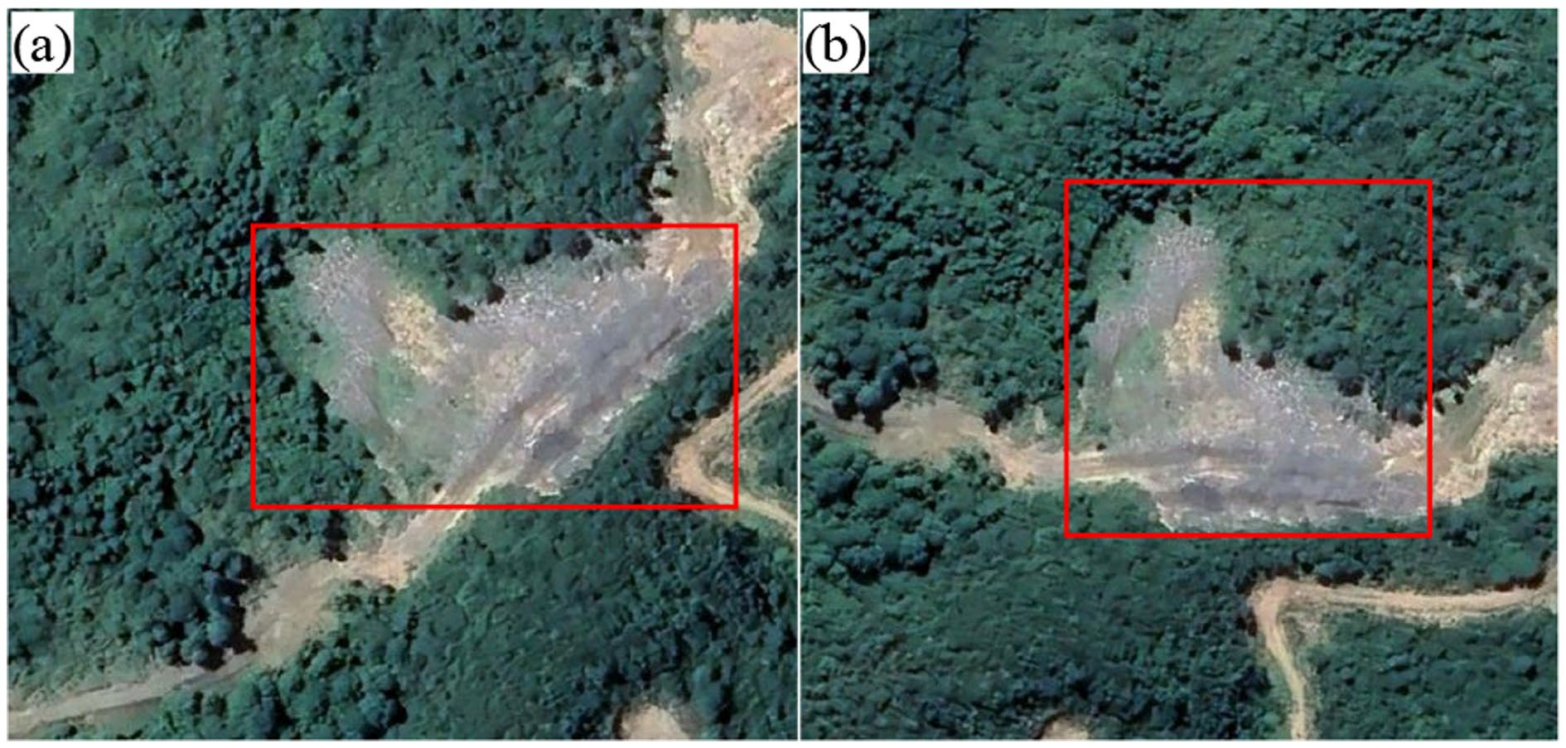
Differences in landslide postures.

*Pose variations*: adjusting landslide orientation via Google Earth’s panoramic view to capture diverse postures (Figure 3). Among them, the differences are marked with red boxes.

For model training and evaluation, the 778 real samples were randomly split into training and validation sets at a ratio of 6:2, ensuring geographic representativeness and minimizing data leakage. To alleviate data scarcity, we further generated synthetic landslide images using a trained Deep Convolutional Generative Adversarial Network (DCGAN). All synthetic samples were added exclusively to the training set, while the validation set remained composed entirely of real, unlabeled landslide instances. This protocol guarantees an unbiased assessment of model performance on genuine geohazard scenarios.

### 3.2 Data augmentation

To increase the number of training samples and enable the model to learn more landslide features, various sample enhancement operations are generally performed on the input original samples during the training process. Traditional data augmentation methods mainly involve simple geometric transformations or color transformations. These data augmentation operations all redistribute or interfere with image pixels. This type of traditional data augmentation method expands the data based on existing data without changing the content of the images themselves. Therefore, the data enhanced by such methods cannot well supplement the spatial distribution of training sample data due to the limitations of manually designed rules. We used the 778 images in Section 3.1 as the basic dataset. Then, by using the Generative Adversarial Network (GAN) method, the model can learn the distribution of the training data and generate new images similar to the training dataset based on this data distribution pattern. We will use the GAN-based method for landslide sample generation to conduct data augmentation for landslide samples. DCGAN is an improved version of the Generative Adversarial Network (GAN), aiming to generate more realistic images through convolutional neural networks. DCGAN introduces deep convolutional networks into the GAN model, using multiple convolutional and transposed convolutional (deconvolutional) layers to extract and generate image features, further improving the quality of generated images.

**Figure 4:**
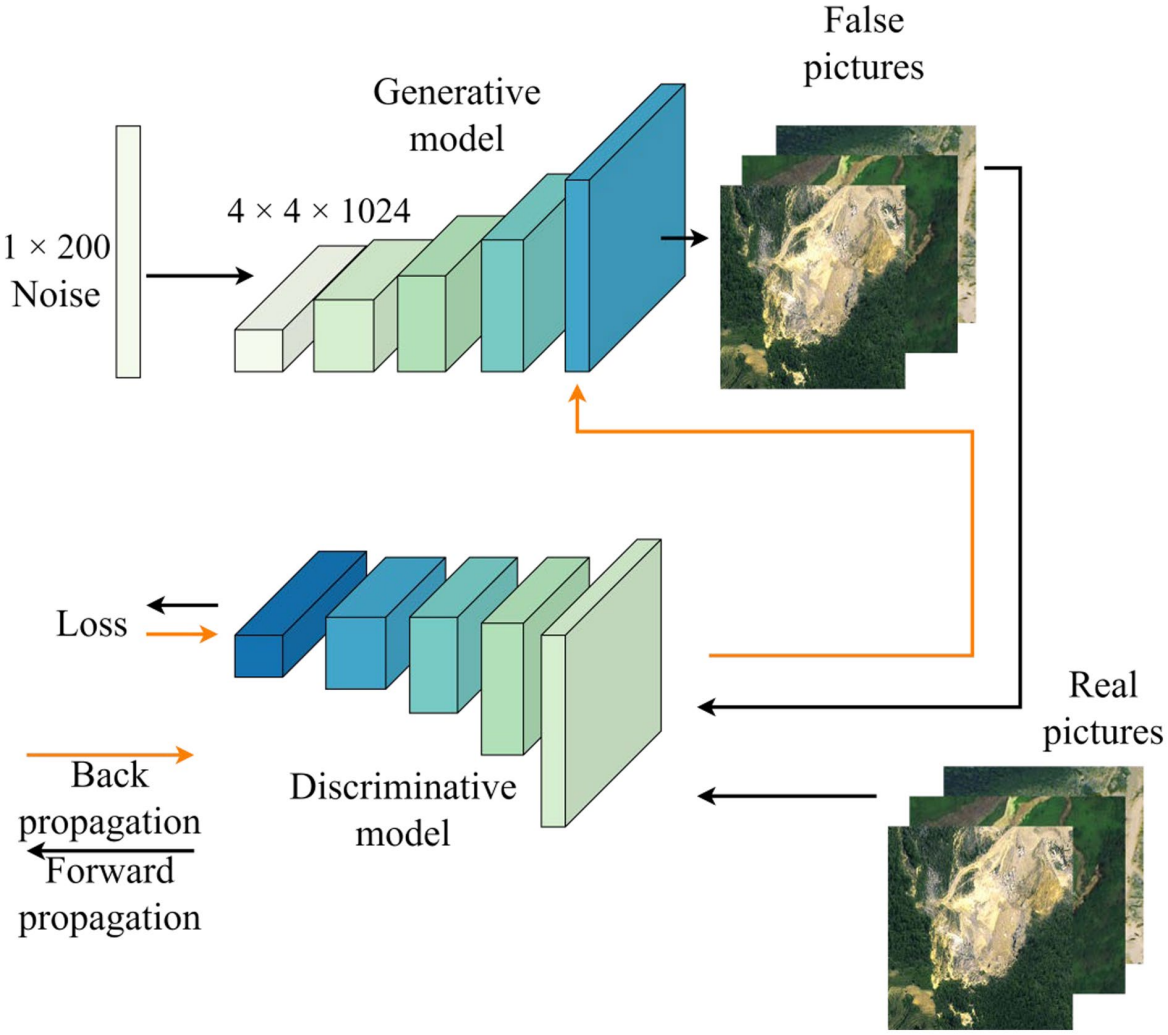
Schematic diagram of the DCGAN model.

The basic structure of the DCGAN model is the same as that of GAN, including two parts: a generator and a discriminator. The generator generates a fake image through input random noise, while the discriminator takes the image generated by the generator as input and judges whether the generated image is real, outputting a probability of 1 if it is real and 0 if it is not. Through continuous training, the images generated by the generator will increasingly resemble real images until the discriminator cannot distinguish between real and fake, at which point the generated images are considered real. Subsequently, we performed difficult sample construction data augmentation, small target sample recombination, and edge sample construction data augmentation on the images generated by DCGAN. Figure 4 shows a schematic diagram of the DCGAN model.

DCGAN and GAN have the following key changes in model structure design:

Convolutional and transposed convolutional layers are used instead of fully connected layers in the GAN structure to achieve feature extraction and image generation through convolutional and transposed convolutional operations.

The DCGAN model uses Batch Normalization (BN) technology in the generator, and the addition of BN layers makes the model training more stable.

The DCGAN model adds the LeakyReLU activation function in the discriminator, which can reduce the occurrence of the gradient vanishing problem.

#### 3.2.1 Difficult sample construction data augmentation

The probability of false detection is reduced by constructing complex samples filled with negative samples similar to landslides in the sample background. Starting from the imagery features of landslides, difficult landslide samples with similar color features, shape features, and spectral features are constructed to enrich the landslide sample dataset.

1. Color feature-similar difficult samples.

The landforms most similar to landslides in terms of color features on remote sensing imagery are mainly bare land, dry land, and some earthen roads. These landforms are extremely likely to be misclassified as landslides by some traditional machine learning algorithms. From the perspective of existing deep learning landslide detection algorithm research, these landforms are still the main targets causing model misclassification. Therefore, in the process of constructing difficult samples, a landslide sample is taken as the main body, and three negative samples such as bare land, dry land, and earthen roads are used as the background to construct a landslide difficult sample with a complex background through random position distribution to improve the accuracy of the landslide detection model.

1. Shape feature-similar difficult samples.

Landslides generally appear in inverted pear-shaped, tongue-shaped, long-chair-shaped, horn-shaped, etc., on remote sensing imagery. Some reclaimed farmland, artificially excavated land, and other landforms also have similar morphological features. Using these negative samples with similar shapes to landslides to form the sample background can construct more interfering landslide difficult samples.

1. Spectral feature-similar difficult samples.

Heterogeneous objects with the same spectrum are one of the main reasons for the difficulty in remote sensing imagery classification, so difficult landslide samples can be constructed using the characteristic of heterogeneous objects with the same spectrum. On remote sensing imagery, factors such as different solar illumination angles, densities, and water contents cause the spectral features of some landslides and certain buildings, concrete hardened roads, and other landforms to appear similar. These similar negative samples are used to enrich the background of landslide samples.

#### 3.2.2 Edge sample construction data augmentation

Remote sensing imagery covers a wide range of landforms, which is also a major advantage of remote sensing detection. However, in the practical application of deep learning remote sensing landslide detection, due to the limitations of computer performance, it is impossible to input the entire scene imagery into the model for detection at once. Generally, the imagery needs to be cropped into image slices suitable for the input size of the network model before being sent to the model. During the cropping process, landslide targets will inevitably be cropped into incomplete targets, causing difficulties for landslide detection and reducing detection accuracy. Therefore, this paper improves the model’s detection accuracy for incomplete targets by simulating the incomplete landslide targets caused by random cropping to construct complex edge samples for model training.

First, two landslide samples are randomly selected from the original samples. Then, these two samples are cut along the horizontal or vertical direction based on the image center point to obtain four cut images. Finally, the cut images are spliced two by two along the cutting line to obtain two new edge samples.

#### 3.2.3 Small target sample recombination

Due to differences in the resolution and shooting perspective of remote sensing imagery, some small-scale landslides appear as very small targets on the imagery. In the training process of the landslide detection model, small target landslides contain less information, resulting in serious missed detection problems in the trained model. By constructing small target training samples, this missed detection problem can be alleviated. Therefore, the construction of small target samples can improve the model’s detection accuracy for small landslides in remote sensing imagery and solve the problem of easy missed detection of small landslides during the landslide detection process. The principle formula for constructing the small target sample is shown in Equations (1–2).

$$\lambda_{i}=\text{random}\;(0.1,0.3)$$ (1)

$$\text{Smal}l_{\text{sample}}=F(\lambda_{i}*\text{Smal}l_{\mathrm{img}})i\in [1,9]$$ (2)

Here, *λ_i_* represents the random scaling coefficient, *Small_sample* denotes the newly concatenated sample, and *F* is the concatenation function.

Drawing on the Mosaic data augmentation idea in YOLOv5 to construct training samples for small objects. Randomly select 9 samples from the original ones to create a complex sample containing multiple small-object landslides. First, randomly select 9 landslide sample images from the original samples and randomly scale the 9 images. The length and width of the scaled images are both less than one-third of those of the original images. Second, place the scaled images sequentially and randomly in each of the nine grids of an original-image-sized tic-tac-toe grid to them into a complex sample. Third, apply the same transformation operations to the landslide labels to obtain the labels after the recombination of small samples.

### 3.3 NL-YOLOv5 model

**Figure 5:**
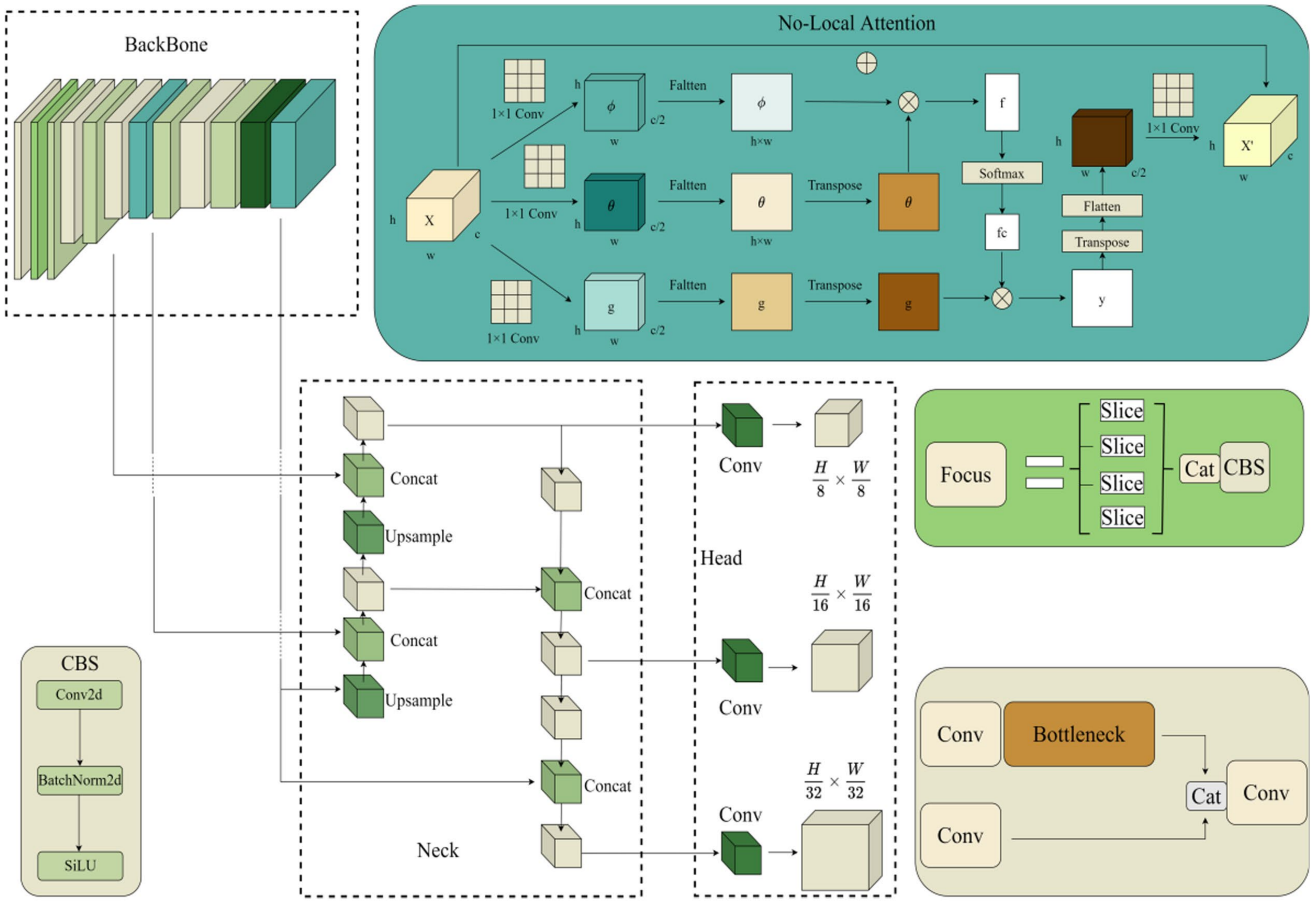
NL-YOLOv5 model.

The specific improvements of the NL-YOLOv5 model include the following two aspects: (1) Taking the object detection algorithm YOLOv5 as the base network, the non-local information statistics attention mechanism is incorporated into the network to reconstruct the YOLOv5 network structure. The aim is to integrate more global information into the network, enabling the model to capture both local and global information and provide richer semantic information for subsequent feature layers, thereby improving the accuracy of landslide detection. (2) Drawing on the idea of ASPP (Atrous Spatial Pyramid Pooling), an improved large-convolution-kernel spatial pyramid pooling module (LK-SPP) is developed to replace the SPPF module in YOLOv5. Considering that the use of large convolution kernels will inevitably bring about greater computational complexity, to balance this, depthwise separable convolutions are utilized instead of regular convolutions during the design of the LK-SPP module. The purpose is to enhance the effective receptive field of the model using large convolution kernels without incurring a huge computational cost. The structure of the NL-YOLOv5 model is shown in Figure 5.

#### 3.3.1 No-local attention module

The No-Local Attention is an attention mechanism that captures temporal, spatial, and spatio-temporal long-range dependencies by borrowing the idea of non-local mean filtering operations in the field of image filtering. Its main objective is to improve the receptive field of the network and its ability to capture global information over long distances. The receptive fields of regular convolutions are mostly 3 × 3 or 5 × 5 in size, whereas the use of No-Local Attention can effectively enlarge the receptive field rather than confining it to a local area. The general Equation 3 for No-Local Attention is as follows:

$$y_{i}=\frac{1}{x(x)}\sum_{\forall j}f(x_{i},x_{j})g(x_{j})$$ (3)

Among them, *x* is the input feature map; *i* represents the response at the current position, and *j* represents the global response. A non-local response value is obtained through weighted summation; the *f* function calculates the correlation between the current position *i* and the global position *j*; the g function computes the result of the feature map at the global position *j*; the final output *yᵢ* is obtained after normalization using the response factor *C*(*x*).

**Figure 6:**
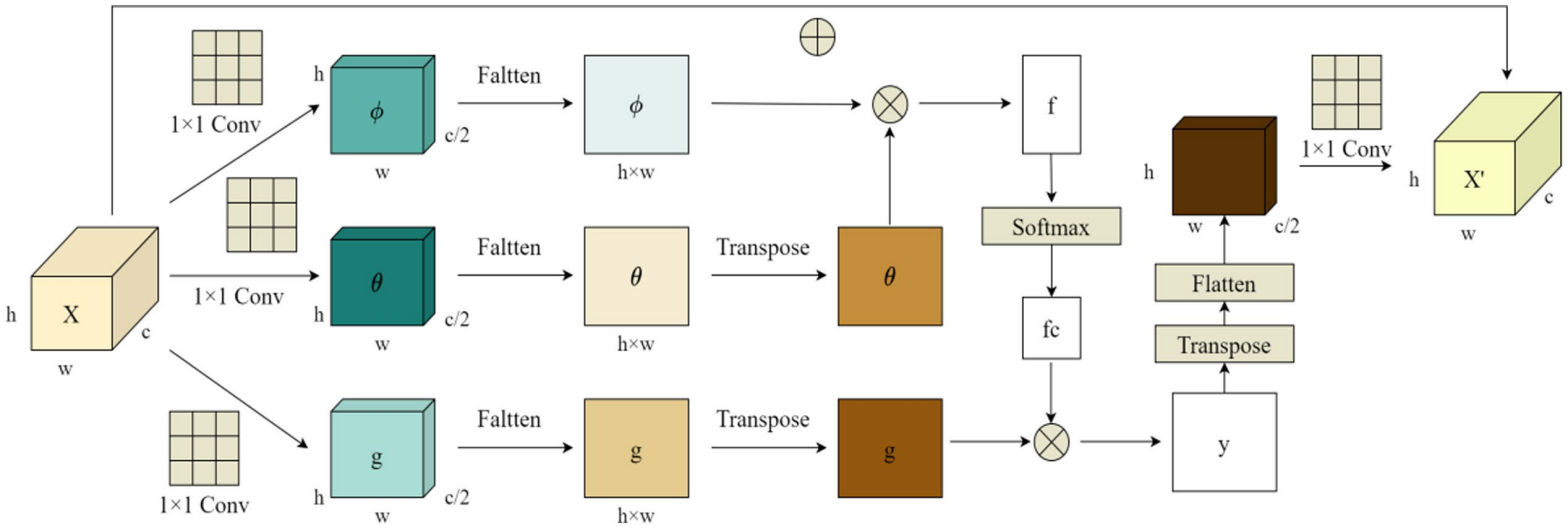
No-local attention module.

The computational flowchart of the No-Local Attention is shown in Figure 6. Assuming the input feature map is *x*, with size [*c*, *h*, *w*], where *c* denotes the number of channels, *h* the height, and *w* the width of the feature map. A 1 × 1 convolution is used for channel compression on the input feature map *x*, yielding feature maps *φ*, *θ*, and *g* with size [*c*/2, *h*, *w*]. Subsequently, *φ*, *θ*, and *g* are unfolded along the *h* and *w* dimensions to obtain *φ*, *θ*, and g tensors of size [*c*/2, h*w]. The *θ* and *g* tensors are transposed to obtain a channel-reordered *θ* tensor with shape [h*w, *c*/2]. Next, matrix multiplication is performed between *θ* and *φ* to compute their correlation, resulting in a tensor *f* of size [h*w, h*w]. The Softmax function is applied to normalize the results, obtaining the attention coefficient tensor *fₖ*. Matrix multiplication is then conducted between *fₖ* and the transposed *g* tensor to yield a feature tensor *y* of size [h*w, *c*/2]. The feature tensor y is sequentially transposed and flattened to obtain a feature tensor of size [*c*/*2*, *h*, *w*]. The feature tensor obtained in the previous step is then passed through a (Feng et al., 2026; Feng et al., 2026; Feng et al., 2026) convolution to expand the channels back to the original number *c*, resulting in a feature tensor *x*’ with the same shape as the input *x*. Finally, a residual connection is applied between *x* and *x*’ to obtain the final feature map.

#### 3.3.2 LK-SPP module

In the context of landslide detection from remote sensing imagery, capturing multi-scale contextual information while preserving fine spatial details is critical. To this end, we adopt a modified spatial pyramid pooling strategy inspired by—but distinct from—the Atrous Spatial Pyramid Pooling (ASPP) module. ASPP utilizes dilated convolutions with varying rates to enlarge the receptive field without spatial downsampling, yet its sparse sampling pattern can lead to discontinuities in feature representation, particularly at high dilation rates where large portions of the input are effectively ignored.

To mitigate this issue in our setting, we replace the dilated convolutions (with rates 12, 18, and 24) in the conventional ASPP design with large-kernel depthwise separable convolutions of sizes 21 × 21, 31 × 31, and 41 × 41, respectively. This choice draws on recent observations that dense, large-kernel convolutions can achieve wide receptive fields while maintaining pixel-wise connectivity—thus better preserving structural continuity in geospatial features such as landslides. The resulting component, which we refer to as Large-Kernel Spatial Pyramid Pooling (LK-SPP), is not proposed as a general-purpose architectural novelty, but rather as a task-informed adaptation tailored to the characteristics of landslide imagery.

**Figure 7:**
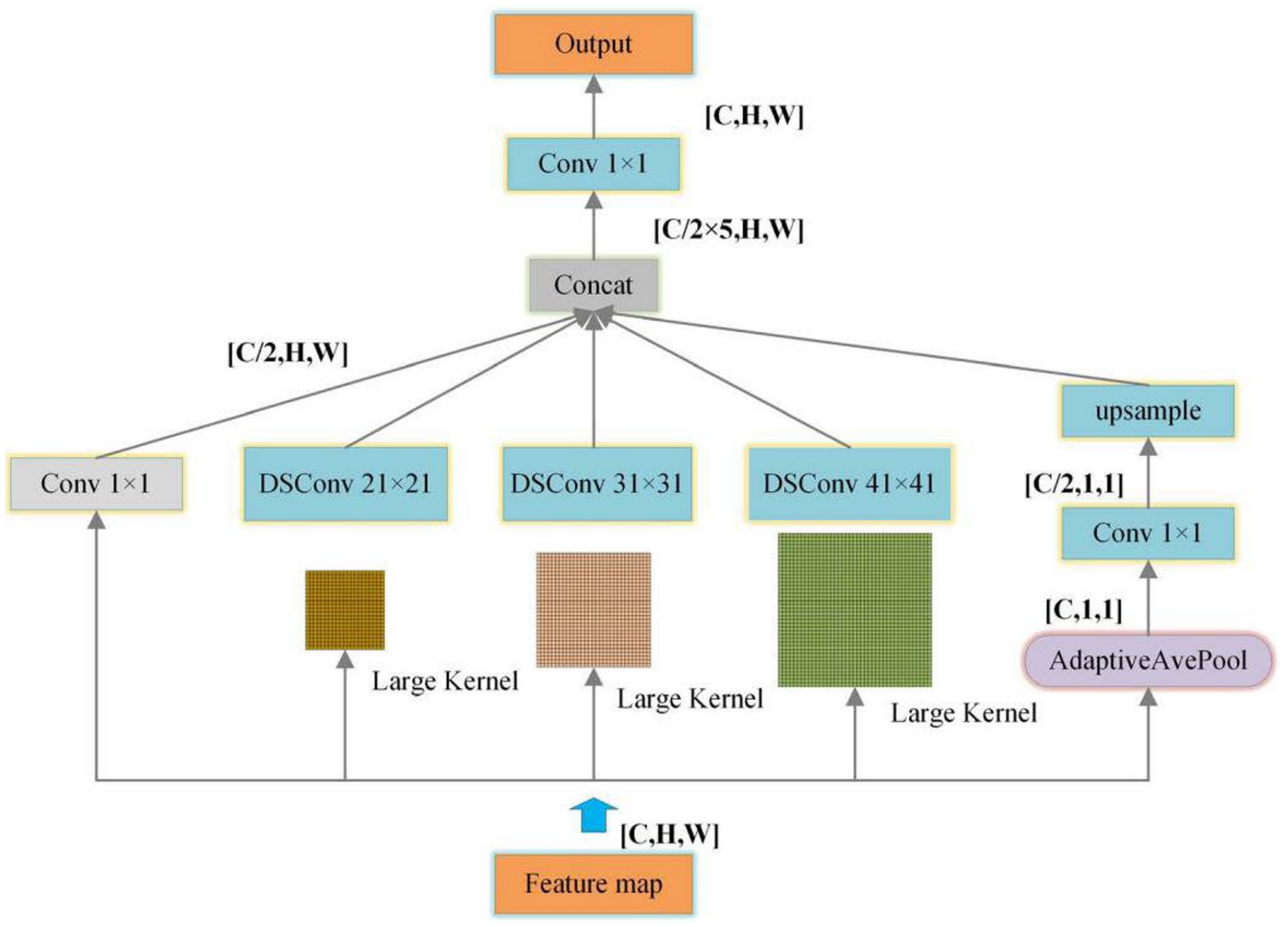
LK-SPP module.

As illustrated in Figure 7, the LK-SPP comprises two parallel pathways. The first pathway processes the input feature map through a 1 × 1 convolution alongside three large-kernel depthwise separable convolutions (21 × 21, 31 × 31, 41 × 41) to extract multi-scale spatial patterns. The second pathway applies adaptive average pooling to capture global context, followed by a 1 × 1 convolution for channel reduction and bilinear upsampling to recover the original spatial resolution. The outputs from both pathways are then concatenated along the channel dimension—yielding five times the original number of channels—and passed through a final 1 × 1 convolution to restore the input channel dimensionality.

This configuration enables the model to integrate both local detail and long-range context in a manner that aligns with the spatial extent and textural complexity of landslide regions, thereby supporting more robust detection performance in our experimental evaluations.

## 4 Experimental results and analysis

### 4.1 Experimental environment

The hardware environment for this experiment includes an Intel Core(TM) i9-9980XE processor, 128GB of memory, and an NVIDIA GeForce RTX 2080 Ti GPU (128GB). The initial learning rate is 10^−2^, the learning rate momentum is 0.937, and the weight decay coefficient is 5 × 10^−4^. During training, the batch size is set to 16, the image resize size is 640, and the model training epoch is set to 100.

### 4.2 Comparative experiments

After training the base model YOLOv5 and the improved NL-YOLOv5 model, landslide data not involved in training were used to test and evaluate the landslide detection performance of the models. The landslide detection accuracy is shown in Table 1.

**Table 1:** Landslide detection accuracy.

Model	*p*	*R*	AP	F1	FPS (f/s)
YOLOv5	0.70	0.77	0.75	0.73	72
NL-YOLOv5	**0.77**	**0.79**	**0.80**	**0.78**	69

The bold text represent the highlighting of the superior values.

As shown in Table 1, compared to the base YOLOv5 model, the NL-YOLOv5 landslide detection model improves the landslide detection accuracy (AP) to 80%, representing a 5% increase. It also achieves 7, 2, and 5% improvements in precision (P), recall (R), and F1 score, respectively. In terms of detection speed, the FPS reaches 69 f/s, which shows a slight decrease compared to the base model but remains well above 30 f/s, still meeting the requirements for real-time detection. Importantly, this speed enables processing of a 640 × 640 image in approximately 14.5 ms, allowing rapid screening of large affected areas—e.g., a 10 km^2^ region (covered by ~250 images at 0.6 m resolution) can be analyzed in under 4 s. Such efficiency is critical for post-disaster emergency response, where timely landslide inventory mapping within the ‘golden hours’ after heavy rainfall can directly support evacuation planning and rescue operations.

**Figure 8:**
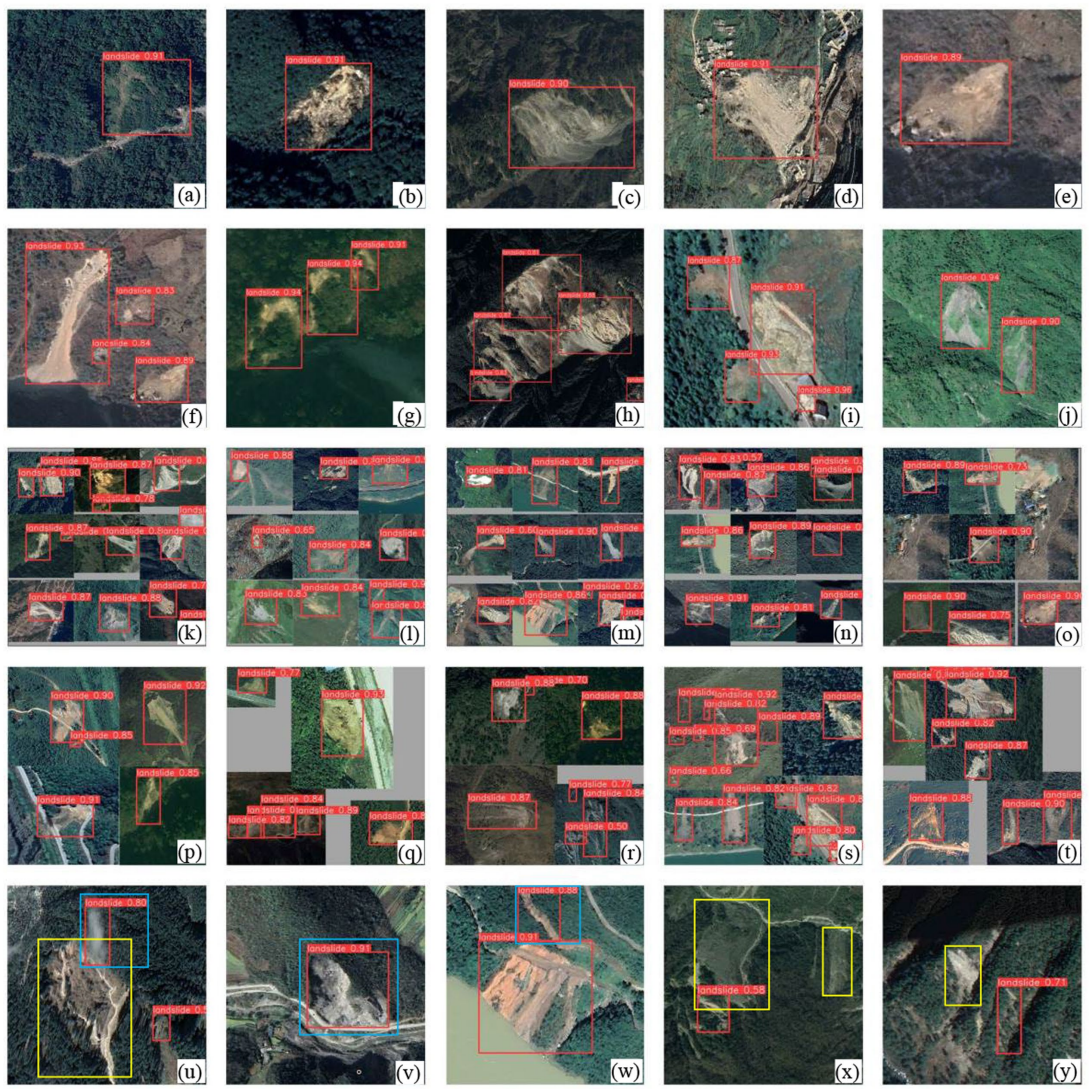
Partial landslide detection results.

Figure 8 presents some landslide detection results, including four groups: single landslide per image, multiple landslides per image, small-target landslides, and mosaic landslides. The small-target landslide test group was designed to evaluate the model’s detection capability for small objects, using test images created by downscaling and stitching some landslide samples from the test set. The mosaic landslides were generated by adopting the mosaic data augmentation idea from YOLOv5, where four random landslide images were stitched together through random scaling and cropping. The purpose was to test the generalization ability of the landslide detection model and its performance in detecting landslides under complex backgrounds. Based on the landslide detection results, NL-YOLOv5 demonstrates strong generalization capability and can detect landslides in various complex environments. Among them, the detected targets are represented by the red boxes.

Figures 8A–E present test results for single-landslide samples with simple backgrounds. It can be observed that the landslide detection confidence exceeds 0.9, and the detection bounding boxes fully encompass the landslide boundaries. Figures 8F–J show test results for multiple-landslide samples in single images. It is evident that NL-YOLOv5 demonstrates effective detection for both large and small targets. For instance, in Figure 8H, NL-YOLOv5 is capable of detecting secondary landslides on large landslide masses. Figures 8K–O,P represent detection results for small-target landslides and landslides under complex background conditions, respectively. The NL-YOLOv5 model exhibits strong detection capability for small-target landslides with minimal missed targets and can still identify landslide locations in remote sensing images under highly diverse and complex background interference. Due to the limited number of landslide samples, ensuring sample richness and diversity is challenging. Consequently, the model may struggle to accurately classify certain terrain features, leading to some false and missed detections, as shown in Figure 8U–Y. The yellow dashed boxes indicate missed landslide detections, while the blue dashed boxes represent false detections. In Figure 8U, clouds are mistakenly identified as landslides, and in Figure 8V, artificially excavated bare land is misclassified as a landslide. From Figure 8X,Y, it can be observed that missed landslides are generally those that occurred a long time ago, with boundaries that are no longer distinct in remote sensing images and show minimal contrast with the surrounding environment due to natural erosion. Considering the false and missed detections, future work should focus on enhancing the diversity of landslide samples and increasing the number of negative samples prone to misclassification in the dataset to improve model robustness.

*Statistical significance verification*: to ensure the reliability of the performance gains, we conducted statistical analysis based on 5 independent training runs (random seeds 1–5). The NL-YOLOv5 model achieved a mean AP of 80.2% ± 1.3% (95% CI: [78.9, 81.5%]), significantly outperforming the baseline (75.1% ± 1.5%) with *p* < 0.01 via two-sample t-test. This confirms that the 5% accuracy improvement is not due to random fluctuations but reflects a robust model enhancement.

### 4.3 Comparative experiments on attention mechanisms

The purpose of attention mechanisms in computer vision is to enable models to ignore irrelevant information in images and focus more on target information. In landslide detection, attention mechanisms help the model disregard complex background information in remote sensing images and concentrate on the boundaries, textures, and deep semantic information of landslide targets. To investigate the impact of different attention mechanisms on landslide model performance, this study conducted comparative experiments on three commonly used attention mechanisms in CNNs: SE (Kumar et al., 2022), ECA (Wang et al., 2020), and CBAM (Woo et al., 2018). The experimental results are shown in Table 2. From the perspectives of network layers and model parameters, the four attention mechanisms result in similar network depths when integrated, with NL-YOLOv5 having the largest parameter count, though the difference is not substantial. In terms of landslide detection accuracy, the NL-YOLOv5 model performs best, achieving an AP value of 0.80 and the highest F1 score of 0.78. Regarding detection speed, NL-YOLOv5 achieves 69 frames per second (FPS) for landslide detection, effectively improving detection accuracy while ensuring real-time performance. The results indicate that the non-local operation operator in No-Local Attention enables the model to capture more global information. Inserting this attention module into the backbone network allows for comprehensive extraction of landslide features, providing richer information for subsequent feature layers, thereby enhancing landslide detection accuracy.

**Table 2:** Comparative experiments on different attention mechanisms.

Model	Network layer	Para (M)	*p*	*R*	AP	F1	FPS (f/s)
NL-YOLOv5-SE	238	10.56	0.76	0.74	0.78	0.75	68
NL-YOLOv5-ECA	234	10.56	0.75	0.71	0.76	0.73	70
NL-YOLOv5-CBAM	254	10.93	0.74	0.74	0.77	0.74	68
NL-YOLOv5	246	11.88	**0.77**	**0.79**	**0.80**	**0.78**	69

The bold text represent the highlighting of the superior values.

### 4.4 Verifying the impact of DCGAN-based landslide data augmentation on landslide detection models

Table 3 presents the statistics of model accuracy before and after data augmentation. All the evaluation metrics reported in the table were calculated on an independent test set that contained only real landslide images. During any stage of validation or testing, no synthetic samples were involved. Through data augmentation, the model accuracy was significantly improved. The average accuracy (AP) increased by 9%, reaching 0.89. The F1 value increased by 4%, reaching 0.82. As can be seen from the table, after data augmentation, the precision P increased by 9%, indicating that the model after data augmentation can more accurately determine whether the targets in the images are landslides. The recall rate R of the model decreased by 1% after data augmentation. This might be due to the fact that although the generated landslide samples are very close to the real samples, from the quantitative analysis results, the FID is 2.67 and the KID value is 0.08. The generated samples and the real samples still have certain errors. The addition of such non-fully consistent generated samples to the model training caused certain interference to the model, resulting in a certain error in recall rate, so the recall rate decreased.

**Table 3:** Model accuracy before and after data augmentation.

Model	*p*	R	AP	F1
NL-YOLOv5	0.77	0.79	0.80	0.78
DCGAN-NL-YOLOv5	0.86	0.78	**0.89**	**0.82**

The bold text represent the highlighting of the superior values.

**Figure 9:**
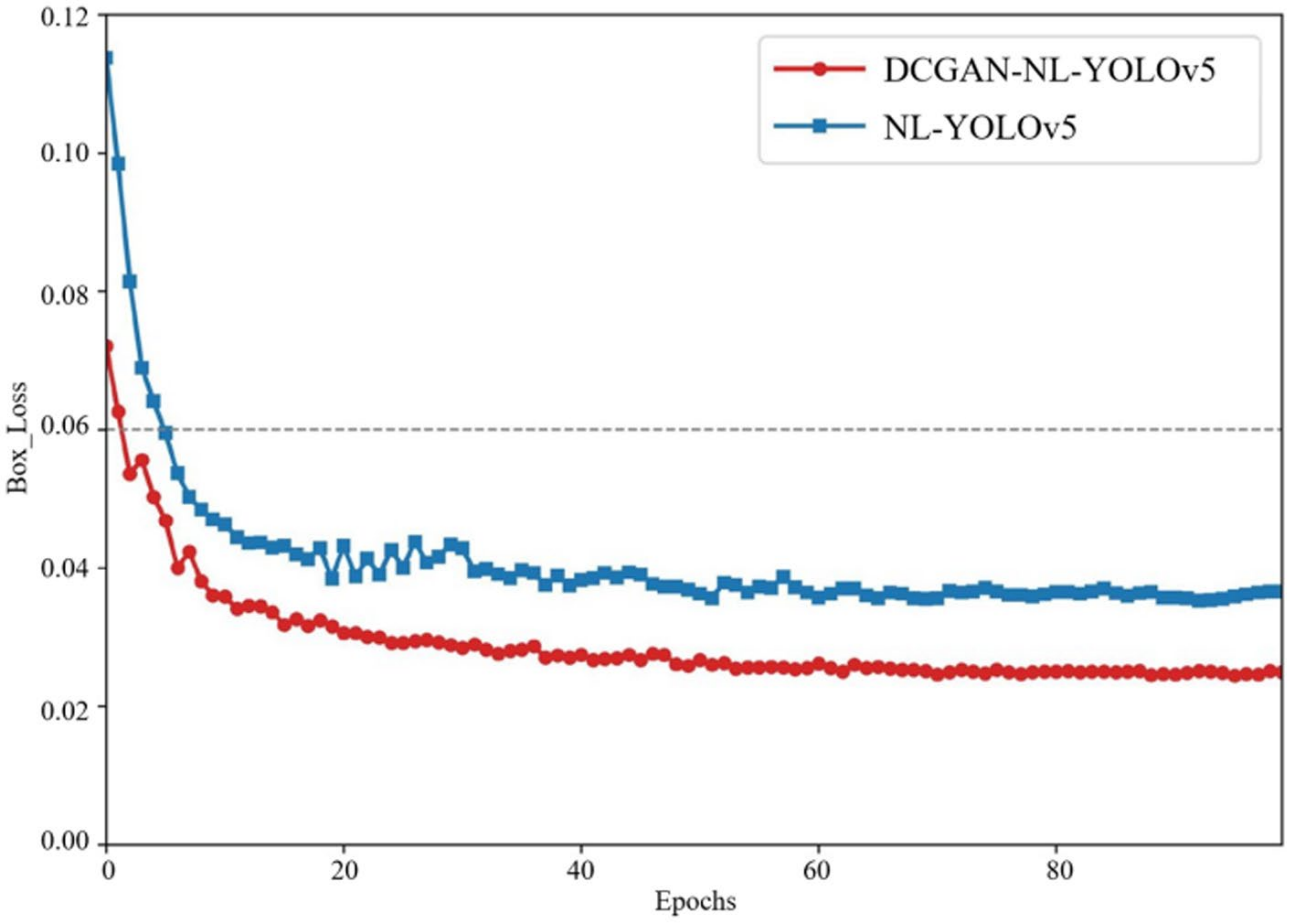
Loss variation curves before and after DCGAN data augmentation.

**Figure 10:**
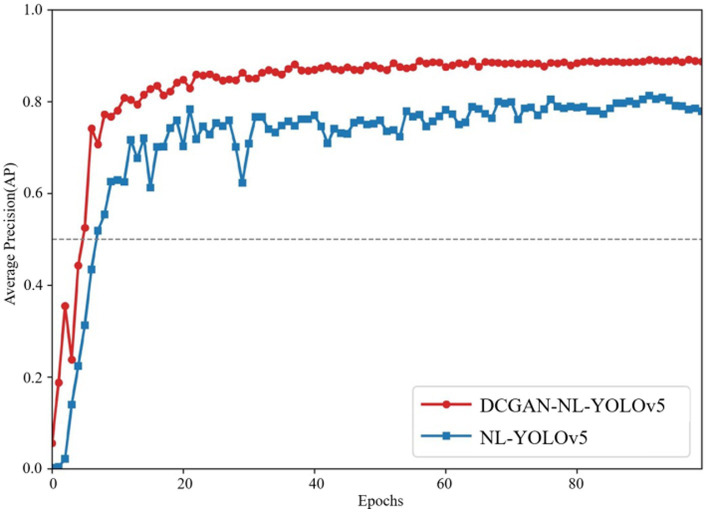
AP variation curves before and after DCGAN data augmentation.

To compare the stability of the model during training before and after dataset optimization, the variations in detection box loss (box_loss) and average precision (AP) with respect to Epoch during the training process were visualized. The visualization results are shown in Figure 9 and Figure 10.

Figure 9 presents the curves of model detection box loss during training before and after data augmentation, while Figure 10 illustrates the changes in model validation AP during training. The blue curve represents the NL-YOLOv5 model, and the red curve represents the DCGAN-NL-YOLOv5 model. As shown in Figure 9, after data augmentation using landslide samples generated by DCGAN, the model converges faster and exhibits a smoother loss curve. This indicates that for deep learning models, larger training datasets better unleash the performance of deep learning models. From Figure 10, it can be observed that the model accuracy after data augmentation is significantly improved, and the AP curve becomes smoother. This demonstrates that enhancing training samples through generative models can effectively enhance model performance and precision.

### 4.5 Impact of different convolutional kernel size combinations in LK-SPP on model performance

In traditional CNN theory, a large convolutional kernel can be entirely replaced by multiple smaller kernels without information loss. For example, a 5 × 5 convolution can be substituted with two 3 × 3 convolutions, and a 7 × 7 convolution can be replaced by three 3 × 3 convolutions. While the theoretical receptive field of stacked smaller kernels matches that of a larger kernel, the effective receptive field of larger kernels is greater. A larger effective receptive field enables the model to perceive more global feature information. Therefore, we designed large convolutional kernels in the LK-SPP module to enhance the model’s ability to capture global information. During experiments, three combinations of convolutional kernel sizes—7-7-7, 21-31-41, and 31-41-51—were tested for the three depthwise separable convolution blocks in the LK-SPP structure. The experimental results are shown in Table 4.

**Table 4:** Impact of different convolutional kernel sizes in LK-SPP on landslide detection accuracy.

Model	Conv kernel size	Para (M)	*p*	*R*	AP	FPS (f/s)
NL-YOLOv5	LK-SPP [7-7-7]	10.37	0.74	0.72	0.76	68
	LK-SPP [21-31-41]	11.88	**0.77**	**0.79**	**0.80**	69
	LK-SPP [31-41-51]	12.98	0.76	0.74	0.78	66

The bold text represent the highlighting of the superior values.

As can be seen from the table, as the convolutional kernel sizes in the LK-SPP module continuously increase, the model’s parameter count also rises, but the increase is marginal, primarily due to the adoption of depthwise separable convolutions. When the convolutional kernel size increases from 7 to 41, the Average Precision (AP) for landslide detection gradually improves, with the LK-SPP [21-31-41] combination demonstrating the best performance, achieving an AP of 0.80. However, when the convolutional kernel size is further increased to 51, the detection accuracy begins to decline. During experiments, it was observed that the landslide detection speed did not exhibit significant fluctuations and consistently met the requirements for real-time detection. Overall, increasing the convolutional kernel size provides a certain improvement in model performance, but larger convolutional kernels also impose a parameter burden on the model. Moreover, based on the experimental results, it is evident that a larger convolutional kernel size is not always better.

## 5 Conclusion

In recent years, deep learning–based approaches have been increasingly applied to landslide detection in remote sensing imagery. However, practical deployment remains hindered by persistent challenges, including limited labeled data, suboptimal detection accuracy, and insufficient model generalization—particularly in data-scarce scenarios. To address these issues in a task-oriented manner, this study presents a systematic integration and empirical evaluation of established deep learning components tailored specifically for landslide detection.

Our work focuses on two complementary aspects:

1. Data augmentation via GAN-based synthesis: Given the scarcity of real landslide samples, we employ a Deep Convolutional Generative Adversarial Network (DCGAN)—a well-established generative framework—to synthesize additional training examples. The trained generator produces images that preserve key visual characteristics of real landslides, such as color distribution, geometric shape, textural patterns, and micro-topographic cues. These synthetic samples are used to enrich the training set, thereby mitigating overfitting and improving model robustness.
2. Task-adapted detector design: Building upon the YOLOv5 architecture, we integrate two existing yet effective modules—Non-Local Attention for global context modeling and a Large-Kernel Spatial Pyramid Pooling (LK-SPP) block for multi-scale feature aggregation—into the backbone network. This configuration, which we refer to as NL-YOLOv5, is not intended as a novel architecture per se, but rather as a purpose-built pipeline that leverages known techniques to better suit the spatial and contextual demands of landslide detection.

Using our curated dataset (augmented with DCGAN-generated samples), we conduct controlled experiments comparing the adapted NL-YOLOv5 against the baseline YOLOv5. Results show that the integrated approach achieves an average precision (AP) of 80%—a 5 percentage point improvement—while maintaining a real-time inference speed of 69 frames per second (fps). These findings demonstrate that thoughtful integration and rigorous validation of existing components, when guided by domain-specific requirements, can yield meaningful performance gains in geohazard monitoring applications.

## Data Availability

Publicly available datasets were analyzed in this study. This data can be found at: https://gpcv.whu.edu.cn/data/Bijie_pages.html.
